# Novel Antimicrobial Approaches to Combat Bacterial Biofilms Associated with Urinary Tract Infections

**DOI:** 10.3390/antibiotics13020154

**Published:** 2024-02-04

**Authors:** Giuseppe Mancuso, Marilena Trinchera, Angelina Midiri, Sebastiana Zummo, Giulia Vitale, Carmelo Biondo

**Affiliations:** Department of Human Pathology, University of Messina, 98125 Messina, Italy; mancusog@unime.it (G.M.); tmarilena08@gmail.com (M.T.); amidiri@unime.it (A.M.); zummo@unime.it (S.Z.); giulia.vitale19@gmail.com (G.V.)

**Keywords:** urinary tract infection, nanoparticles, multidrug resistance, alternative strategies

## Abstract

Urinary tract infections (UTIs) are prevalent bacterial infections in both community and healthcare settings. They account for approximately 40% of all bacterial infections and require around 15% of all antibiotic prescriptions. Although antibiotics have traditionally been used to treat UTIs for several decades, the significant increase in antibiotic resistance in recent years has made many previously effective treatments ineffective. Biofilm on medical equipment in healthcare settings creates a reservoir of pathogens that can easily be transmitted to patients. Urinary catheter infections are frequently observed in hospitals and are caused by microbes that form a biofilm after a catheter is inserted into the bladder. Managing infections caused by biofilms is challenging due to the emergence of antibiotic resistance. Biofilms enable pathogens to evade the host’s innate immune defences, resulting in long-term persistence. The incidence of sepsis caused by UTIs that have spread to the bloodstream is increasing, and drug-resistant infections may be even more prevalent. While the availability of upcoming tests to identify the bacterial cause of infection and its resistance spectrum is critical, it alone will not solve the problem; innovative treatment approaches are also needed. This review analyses the main characteristics of biofilm formation and drug resistance in recurrent uropathogen-induced UTIs. The importance of innovative and alternative therapies for combatting biofilm-caused UTI is emphasised.

## 1. Introduction

Approximately 15% of antibiotics are prescribed for the treatment of urinary tract infections (UTIs), which are some of the most common bacterial infections. UTIs affect over 400 million people annually and result in 150 million deaths worldwide [1,2]. It is estimated that around 50% of women and 5% of men will experience a UTI during their lifetime [3]. The incidence of UTIs increases with age, affecting between 30% and 50% of women over the age of 60 [4]. UTI pathogenesis is the result of several complex interactions between a uropathogen and the host [5,6]. However, it is widely recognised that outpatient antibiotic use is often excessive and unnecessary [7]. While antibiotics have been used to treat common infections for many years, antibiotic resistance has made several antibiotics that are commonly used for UTIs ineffective, leading to more serious illness, hospital admissions, and deaths, as well as increased healthcare costs [6,8]. Antibiotic resistance is a major concern, with antibiotic-resistant UTIs being a critical aspect [9]. Antibiotic resistance was identified by the World Health Organization as one of the top ten global public health threats in 2021 [10]. A urinary tract infection that spreads to the bloodstream and causes sepsis can be fatal, and drug-resistant infections can make this more likely [6,11]. In 2019, drug-resistant infections directly caused 1.27 million of the approximately 4.95 million deaths attributed to antibiotic resistance. This exceeded the number of deaths caused by HIV/AIDS [12,13]. Antibiotic resistance is a natural phenomenon, but its acceleration due to the misuse of antibiotics in humans and animals is a serious problem [14]. This is especially concerning for UTIs, which are among the most prevalent infectious diseases [15]. Biofilms are widely recognised as a major contributing factor of the high rates of recurrence and antibiotic resistance that are commonly associated with UTIs [16,17]. Biofilms can be formed by different types of bacteria, including both Gram-positive and Gram-negative species, and they are known to play an important role in several disease processes [18,19]. This review analyses the significance of biofilm formation in recurrent UTIs and summarises recent developments in understanding how drug resistance evolves in major uropathogens. The importance of rational antibiotic use in UTI treatment and the factors contributing to increasing bacterial resistance are also highlighted. It also assesses the advantages and disadvantages of alternative treatments for UTIs.

## 2. Classification and Pathogenesis of UTIs

### 2.1. Types of UTIs

There are several classification systems for UTIs [20]. However, the most widely used systems are those developed by the CDC, which distinguish between uncomplicated and complicated UTIs on the basis of the presence of risk factors such as anatomical or functional abnormalities in the urinary tract [5,6,21]. UTIs are also classified according to the location of the infection and the clinical presentation of the UTI [6]. The urinary tract consists of the kidneys, ureters, bladder, and urethra. The kidneys filter waste products from the blood and produce urine, which then passes through the ureters to the bladder. UTIs are typically categorized as upper or lower depending on their location within the urinary tract. Urethritis is an inflammation of the urethra, and ureteritis is an inflammation of the ureter [22]. Lower UTIs, including cystitis, which refers to a bladder infection, and upper UTIs, such as pyelonephritis involving the kidney, are classified based on the affected area [23]. In healthy, pre-menopausal, non-pregnant women with no history of an abnormal urinary tract, acute cystitis and pyelonephritis are the classifications for uncomplicated UTIs, with women being more commonly affected than men [24]. Complicated UTIs involve functional or metabolic abnormalities, such as obstruction, urolithiasis, pregnancy, diabetes, neurogenic bladder, renal, or other immunocompromising conditions [25]. Recurrence, catheter association, and urosepsis are also included in the classification of UTIs [26]. UTIs are classified as recurrent if two or more episodes occur within six months or three or more episodes occur within 12 months [27,28]. Catheter-associated urinary tract infections (CAUTIs) account for around 75% of all hospital-acquired UTIs [29]. The risk of contracting these infections increases with prolonged use of a urinary catheter, which is a tube inserted into the bladder through the urethra to drain urine. Urosepsis is a systemic inflammatory response to UTIs such as cystitis, bladder infection, and pyelonephritis, and it can lead to multiple organ dysfunction, failure, and even death [30].

### 2.2. Clinical Syndromes

Diagnosing UTIs can be challenging, especially in patients with non-specific symptoms [31]. However, it is crucial to differentiate between UTIs and asymptomatic bacteriuria to determine the necessity of antibiotic treatment [6]. Asymptomatic bacteriuria is the presence of one or more bacterial species in urine, as indicated by a positive urine culture, in a patient who does not exhibit any signs or symptoms of a UTI (see below) [32]. In contrast, UTIs are infections that can affect the urethra, bladder, ureters, and/or kidneys. The symptoms experienced depend on which part of the urinary tract is affected [4,32]. Table 1 shows the signs and symptoms of UTIs depending on the location of the bacterial infection.

**Figure 1:**
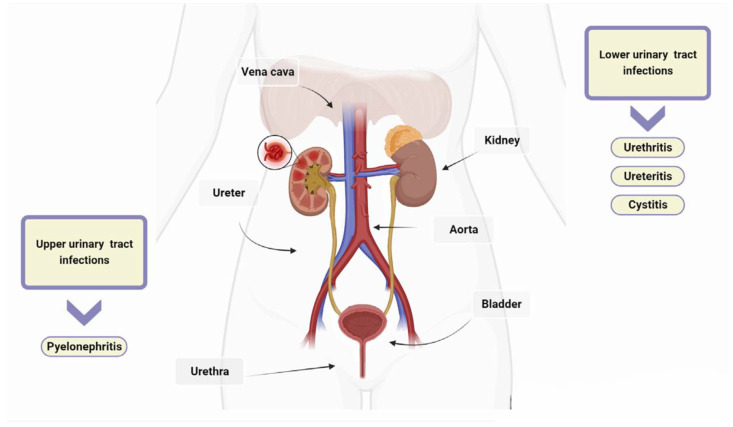
Urinary tract infections (UTIs). UTIs are classified as upper or lower based on their anatomical location. Lower UTIs are further categorised by the specific anatomical part affected, such as urethritis for the urethra, ureteritis for the ureter, and cystitis for the bladder. If microorganisms evade the host’s defences and are not promptly eradicated, they can travel from the lower urinary tract to the kidneys and, if left untreated, can lead to pyelonephritis.

UTIs are more common in women and typically affect the bladder and urethra [18]. Uropathogens commonly infect the urinary tract through an ascending route that starts in the genital area, passes through the urethra to the bladder, and then ascends the ureters to reach the kidneys (Figure 1). Cystitis is a prevalent UTI among women, especially pregnant women, due to pregnancy’s interference with bladder emptying [33]. Women are more susceptible to cystitis due to the shorter length of their urethra and its proximity to the anus. Recurrent episodes of cystitis are common among women, especially during their reproductive years, as bacteria can travel from the urethra to the bladder during sexual intercourse [34]. Bladder infections in women can also be caused by the use of a diaphragm for contraception or the decrease in estrogen production after menopause [35]. The spermicide used in conjunction with a diaphragm suppresses the natural vaginal flora, allowing the bacteria responsible for cystitis to thrive [21]. Bladder prolapse can occur as a result of decreased estrogen production during menopause, which can make it difficult to empty the bladder and increase the risk of bladder infections [36]. In men, cystitis and urethritis are frequently caused by bacterial prostatitis resulting from ascending urethral infection or intraprostatic reflux [37]. If antibiotics fail to eliminate all bacteria, such as when they do not penetrate the prostate adequately or when treatment is discontinued prematurely, prostatitis may recur and become chronic [38]. Bacteria that persist in the prostate gland have a tendency to re-infect the bladder. Cystitis can also result from a urethral stone obstructing urine flow, preventing the removal of residual bacteria that can rapidly multiply in the bladder, or from a catheter that introduces bacteria into the bladder [4]. Urethritis is an infection of the urethra, which is the tube that carries urine from the bladder to the outside of the body. Symptoms of urethritis typically include pain during urination, a strong urge to urinate, and sometimes discharge (which is more common in men than women) [39]. The most common causes of urethritis are sexually transmitted microorganisms, such as *Neisseria*, *Chlamydia*, *Herpes simplex virus*, and *Trichomonas* [40]. Pyelonephritis is a type of urinary tract infection (UTI) that typically originates in another part of the urinary tract, such as the urethra or bladder, and then spreads to one or both kidneys [41]. In rare cases (about 5%), infections can also spread to the kidneys from other parts of the body through the bloodstream [4]. Pyelonephritis is more common in women than in men and may be milder and more difficult to recognize in children and the elderly [41]. Risk factors of pyelonephritis include kidney stones, catheterization, urinary anatomical abnormalities, reflux, diabetes mellitus, enlarged prostate, and pregnancy [6].

### 2.3. Urinary Tract Infections Caused by Bacteria

**Figure 2:**
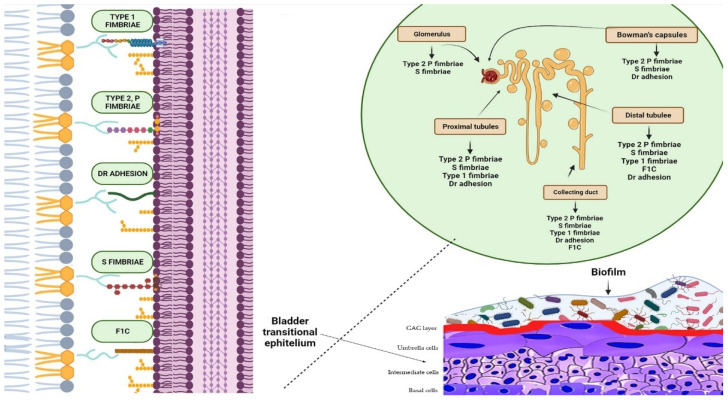
Biofilms are bacterial formations that occur on urothelial surfaces. The spread and colonisation of uropathogens is facilitated by several virulence factors, including adhesion proteins. The figure displays bacterial adhesins that bind to specific host cell membrane structures, such as glycosphingolipids, sialic acid-containing receptors, and glycolipid and mannose receptors, which are expressed in different tracts of the uroepithelium. Type 1 fimbriae facilitate bacterial adhesion at the proximal and distal tubules, as well as at the level of the collecting duct. Bacteria expressing type 2 and S fimbriae can adhere to various parts of the urinary tract, including the glomeruli, Bowman’s capsule, collector duct, and proximal and distal tubules. Adhesion is also possible at the level of the bladder, collecting ducts, and distal and proximal tubules through Dr adhesins. Furthermore, bacteria that are able to colonise the bladder, distal tubule, and collector duct express the adhesion protein F1C, which can result in the formation of biofilms.

UTIs are caused by both Gram-negative and Gram-positive bacteria [42]. Uropathogenic *Escherichia coli* is the most common pathogen found in both uncomplicated and complicated UTIs. This bacterium is responsible for at least 80% and 65% of community- and hospital-acquired UTIs, respectively [6]. For several decades, it was believed that the bladder and urethra of healthy individuals were sterile or contained too few bacteria to cause infections [43]. However, recent studies have shown evidence of numerous microorganisms in the bladder of adult humans without clinical infections. The relevance and contribution of these microorganisms to health and disease are still under investigation [43,44]. Common pathogens causing UTIs include *Enterococcus faecalis*, *Proteus mirabilis*, *Klebsiella pneumoniae*, *Staphylococcus saprophyticus*, *Group B Streptococcus*, *Pseudomonas aeruginosa*, and *S. aureus* [6]. Uropathogens express fimbrial adhesins that bind to glycolipids and glycoproteins on the mucosal–epithelial surface of the host (Figure 2) [45]. Adhesins are used by uropathogens to create biofilms on both biotic and abiotic surfaces, enabling them to colonise the mucosa of the urinary tract and indwelling devices such as urinary catheters [46]. Furthermore, some pathogens secrete substances, such as hemolysin, capsule, proteases, and phospholipases, which aid bacterial invasion by disrupting epithelial integrity [45,47,48,49,50]. Additionally, certain uropathogens, such as UPEC, can invade the host’s epithelial cells and replicate within them, creating a reservoir for recurrent infections [51]. This enables the bacteria to colonise and cause UTIs. Bacterial virulence factors, such as fimbriae, adhesins, P and type 1 pili, and others that facilitate bacterial invasion, have been described in detail in previous comprehensive reviews (Figure 2) [4,6,45,52].

## 3. Biofilm Formation

Bacterial biofilms are a significant cause of UTIs, accounting for around 65% of nosocomial infections and 80% of all microbial infections [53,54]. Biofilms are communities of microorganisms that permanently attach to biotic or abiotic surfaces and are embedded in their own extracellular matrix [55]. This matrix, known as exopolysaccharides (EPSs), consists of several compounds, including polysaccharides, lipids, extracellular DNA, and proteins [56]. The formation of a matrix causes changes in growth rate and gene transcription, resulting in an altered phenotype compared to that of the planktonic counterparts [57]. EPSs play a crucial role in biofilm formation, although their composition varies depending on the bacteria forming the biofilm [58]. They maintain the functional and structural integrity of biofilms, prevent the diffusion of antibiotics to the cell surface of microbes, and protects bacteria in the biofilm from various environmental stresses [59]. Biofilm formation is a multistep process involving reversible and irreversible attachment, the production of EPSs, biofilm maturation, and detachment [60]. The first step in bacterial adhesion is determined by attractive or repulsive forces, such as hydrophobicity, electrostatic interactions, and van der Waals forces [61]. At this stage, planktonic bacteria attach to the surface in a reversible manner, with adhesion being mainly influenced by the surface properties of the substrate [60]. Reversible attachment is typically followed by irreversible attachment, which is mediated by physical appendages of the bacteria, such as fimbriae and pili [62]. This attachment marks the beginning of the evolution of microbes as a biofilm. At this stage, the adhesion process is strengthened by producing EPSs, which facilitate aggregation and adhesion, enabling better surface colonisation. The EPS composition varies among organisms [63]. For example, *E. coli*’s matrix is primarily composed of colanic acid, while *P. aeruginosa*’s matrix produces alginate as its main capsular polysaccharide [64]. Once bacteria have irreversibly attached to a surface, the biofilm begins to grow and mature [18,60]. The ability of a bacterial biofilm to grow is primarily determined by quorum sensing and is limited by factors such as waste removal and nutrient availability in the surrounding environment [65]. The biofilm grows and matures until it reaches a critical mass, which is characterized by a defined three-dimensional structure, water channels, and considerable thickness [18]. Once mature, microbes detach from the biofilm and return to a planktonic state, enabling them to colonise other surfaces and initiate a new biofilm cycle [66]. Different bacteria use various mechanisms to disperse biofilms, which is a crucial step in their transmission both between and within hosts, facilitating the spread of infection [18,46]. Biofilms are also present inside host cells, where they play a vital role in the development of recurrent urinary infections by creating intracellular bacterial communities (IBCs) that shield bacteria from neutrophils and antibiotics [67]. These structures, which are responsible for chronic cystitis, have been extensively described in uropathogenic *E. coli* (UPEC) [67]. Biofilms significantly impact human healthcare, as their formation on medical devices is often linked to persistent infections and tolerance to antibiotic agents [46]. Additionally, medical-device-associated infections are among the leading causes of nosocomial infections [68]. Bacterial biofilms have a significant impact on the development of UTIs, especially in cases of catheter-associated UTIs (CAUTIs), which account for 40% of all hospital-acquired infections [69]. Biofilms can be developed on the urothelium, prostate stones, and implanted biomedical devices by both Gram-positive and Gram-negative bacteria [46]. The most commonly reported bacteria causing UTIs include *E. coli*, *P. mirabilis*, *K. pneumoniae*, *S. epidermidis*, *S. saprophyticus*, *S. aureus*, and *E. faecalis* [18]. *P. mirabilis* is a bacterial pathogen that frequently causes both uncomplicated and complicated UTIs, including CAUTIs. The pathogen can form biofilms because of virulence factors such as fimbriae, capsules, and ureases [70]. Urea hydrolysis leads to the formation of ammonium ions, which cause the precipitation of magnesium and calcium phosphate crystals [70]. The formation of bacterial biofilms protects bacteria from antibiotic compounds that are used to coat catheters. The presence of bacterial biofilm on the urothelium can facilitate bacterial invasion of the renal tissue, leading to pyelonephritis [6]. Biofilm formation also increases the ability of strains to persist in the prostatic secretory system, leading to recurrent UTIs [71]. Previous studies have shown a clear link between recurrent UTIs and biofilm-producing isolates [71]. In healthy women, biofilm formation by UPEC in the vaginal reservoir has been linked to the majority of recurrent UTIs [72]. Biofilms are known to be more resistant to antibiotic treatment than planktonic bacterial cells, which poses a challenge for removing biofilm from devices [16].

## 4. The Role of Biofilm in the Persistence and Recurrence of UTIs

Biofilm cells have distinct characteristics compared to those of planktonic cells, including increased antibiotic resistance and evasion of the innate and adaptive immune response [46]. These characteristics arise from the unique structure of biofilms and the activation of various processes. The high viscosity of the biofilm obstructs the host’s immune defence system and antimicrobial treatments, contributing to its resistance and persistence [73]. Furthermore, the release of extracellular toxins results in significant tissue damage, leading to an increase in nutrient availability and further consolidation of the biofilm [74]. Asynchronous microbial growth within the biofilm gives rise to variant bacterial phenotypes, such as persister cells, which are inherently resistant to antibiotics [60]. Biofilm-forming bacteria exhibit phenotypic variations, which increase the rate of horizontal gene transfer (HGT). HGT can occur through several mechanisms, including conjugation, transformation, and transduction [75]. It is important to note that while these mechanisms are well known, they are not the only ones. Recently, a fourth mechanism has been identified, which involves membrane vesicles that transfer antibiotic resistance genes between members of the biofilm community [76]. CAUTIs caused by bacterial biofilms are a common type of hospital-acquired infection [69]. Urinary catheters are drainage tubes made of silicone or latex that are inserted into the bladder to collect urine. They are commonly used during or after surgery to reduce overflow incontinence or to relieve urinary retention [69]. *E. coli*, *K. pneumoniae*, and *P. mirabilis* are the most common causes of CAUTIs [77]. Bacteria attach to the surface of the catheter and produce urease, which hydrolyses urea into ammonium ions [78]. This process raises the pH of the urine, resulting in the formation of crystals of magnesium and calcium phosphate. These crystals become part of the growing biofilm, which protects the bacteria from compounds used to coat catheters [79]. Previous studies have shown that patients may develop UTIs within a few days of catheterization due to the presence of biofilm on urinary catheters [26,69]. Additionally, it has been found that patients who undergo catheterization for more than 28 days are at a high risk of developing CAUTIs [17]. Biofilms formed in the initial stages of CAUTIs are often colonized by a single species, and subsequently, mixed communities develop, resulting in a thick biofilm that makes antibiotic therapy ineffective [46]. CAUTIs are often caused by Gram-negative bacteria, such as *E. coli* and *K. pneumoniae*, originating from the patient’s perineal microbiota or from the hands of medical professionals [80]. These bacteria can contaminate the urethra and migrate to the bladder, where they create biofilms that act as a reservoir of infection and promote antibiotic resistance. Catheters increase the risk of UTIs because they promote bacterial adherence by damaging the protective mucopolysaccharide layer of the uroepithelium, making it more vulnerable to bacterial invasion [26]. Recent research has shown that enhancing care practices can reduce the incidence of CAUTIs and their associated negative outcomes, including higher costs, longer hospital stays, and increased mortality rates, particularly in critical care units [81,82].

## 5. Resistance of Bacteria in Biofilm

**Figure 3:**
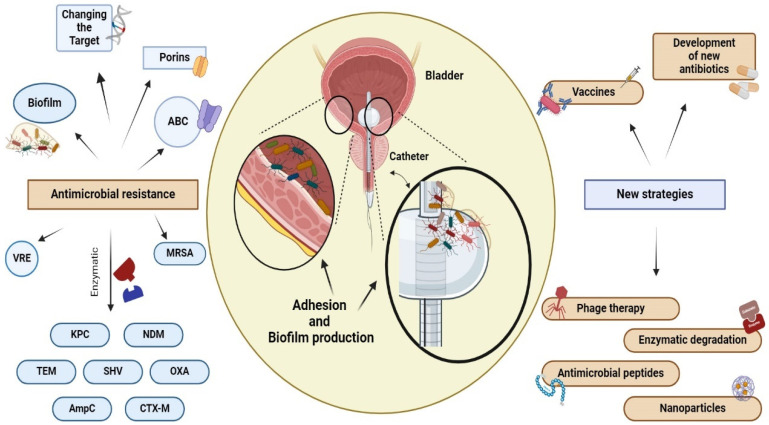
Antibiotic resistance and new therapeutic strategies. Bacteria can develop resistance to antimicrobial drugs through a variety of mechanisms, including the use of hydrolytic enzymes to inactivate the drug, alteration of the drug target through mutation, increased expression of ABC transporters to efflux the drug from the cell, and the formation of biofilms by uropathogens on the surface of the urothelium and on fixed devices, such as catheters. Mechanisms used by VAN and MRSA involve target site alteration, while TET, VIM, and IMP are associated with enzymatic degradation. Additionally, uropathogenic bacteria employ various methods of decreasing the effectiveness of antimicrobial agents. These methods include the production of a thickened cell wall, which makes it difficult for vancomicin to enter the cell (VISA), the use of efflux pumps, and the modulation of porin channels. The presence of biofilm increases the persistence of microorganisms and their antimicrobial resistance. Antibiotics alone may not always be sufficient to eradicate biofilm. Therefore, alternative treatments such as phagotherapy, enzymatic degradation, the use of antimicrobial peptides and nanoparticles, the development of new antibiotics, and vaccines are currently under investigation. NDM: New Delhi Metallo β-lattamasi, KPC: Carbapenemase-producing *Klebsiella pneumoniae*, TEM: β-lactamase isolated from a blood culture from a patient named Temoniera in Greece, SHV: sulfhydryl variant of TEM, OXA: oxacillinase, AmpC: cephalosporinase, CTX-M: cefotaximase, VRE: vancomycin-resistant *Enterococcus*, MRSA: Methicillin-resistant *Staphylococcus aureus*, ABC: ATP-binding cassette.

Antibiotic resistance may be significantly higher in uropathogens that reside in biofilms, up to 1000 times higher than in planktonic bacteria [83]. Biofilms produced by uropathogens play a crucial role in antibiotic resistance through several mechanisms (Figure 3). (1) Antibiotics diffuse inadequately within the biofilm due to the extracellular matrix, causing delayed penetration and reduced effectiveness; (2) bacteria within biofilms exhibit increased expression of efflux pumps compared to that of planktonic cells, which contributes to their resilient resistance to antibiotics; (3) the close cell-to-cell contact within biofilms facilitates the transmission of antibiotic resistance genes through HGT [16,83]. HGT facilitates the transfer of transposons and other mobile genetic elements between biofilm-forming cells, leading to the dissemination of resistance markers [75]. These markers encode the secretion of antibiotic-inactivating enzymes and other virulence factors. (4) The presence of slow-growing persister cells that are intrinsically resistant to antibiotics represents a reservoir of surviving cells capable of rebuilding the biofilm population [84]. The effectiveness of antibiotics is often limited by various mechanisms within a biofilm, which, in turn, drives antibiotic resistance, a topic that has been extensively investigated in many previous studies [6,16,46].

## 6. Strategies for Combatting Biofilm-Forming Pathogenic Microorganisms in UTIs

Given the high levels of antibiotic resistance and the significant contributions to pathogenicity made by microbial biofilms, particularly those formed on catheters in hospitalised patients, there is an urgent need to develop strategies for inhibiting uropathogenic biofilms in UTIs [69]. Although antibacterial drugs can often stop early biofilm formation, once established, biofilms are difficult to eradicate with traditional antibiotic treatments [66,85]. In recent years, a variety of natural and synthetic agents, as well as nanotechnology-based approaches, have been used in clinical settings to disrupt mature biofilms [86]. However, a search of PubMed databases from January 2014 to December 2022 using the keywords ‘phytochemicals’ and ‘anti-biofilm’ did not reveal any approved antibiofilm agents for the treatment of infectious diseases. The most promising agents are still in preclinical development because of their reduced efficacy in in vivo tests. The following section discusses the most promising strategies for eradicating biofilms.

### 6.1. Effectiveness of Antimicrobial Peptides (AMPs) against Biofilm Formation

Antimicrobial peptides (AMPs) are small peptides that exhibit antibiofilm activity against both Gram-positive and Gram-negative bacteria that cause UTIs [87]. Several peptides have been shown to prevent bacterial biofilm formation through various mechanisms, including depolarisation and the subsequent disruption of cell membranes, which allows better penetration of biofilm-inhibiting agents [85]. AMPs not only disrupt membranes but also inhibit biofilm formation and adhesion by degrading extracellular polymeric substances, disrupting cell communication, and downregulating genes responsible for biofilm formation [88]. The antimicrobial properties of AMPs against various bacteria have been extensively studied. Table 2 presents the most well-defined antimicrobial properties of AMPs against different Gram-positive and Gram-negative bacteria.

### 6.2. QS Inhibitors

Quorum sensing (QS) is a cell-to-cell signalling mechanism that enables bacteria to release small signalling molecules called autoinducers that regulate gene expression involved in virulence and biofilm formation [98]. Several QS inhibitors of both natural and synthetic origins have been identified. Blocking QS receptors through the downregulation of the autoinducer-synthetase-encoding gene is one mechanism through which a large group of inhibitors, such as glyptins, exert their activity [99,100]. Phytochemicals from traditional medicinal plants, such as terpenoids, phenols, essential oils, alkaloids, polyacetylene, and lectins, are among the antibiofilm agents that act by blocking the quorum-sensing inducers of bacteria (Table 3) [101,102]. 

However, bacteria can develop resistance to phytochemicals through various mechanisms. Therefore, these products are effectively used in combination with traditional antibiotics to eradicate microbial biofilms [102].

### 6.3. Biofilm Inhibition by Nanoparticles

Nanoparticles, which are less than 100 nm in size, exhibit unique biological activities and have been reported to be effective in treating biofilm-resistant UTIs [109]. Due to their small size, nanoparticles can penetrate cells and deposit ultra-thin coatings on various materials. Previous studies have shown that silver (Ag), nickel (Ni), zinc oxide (ZnO), gold (Au), and copper (Cu) nanoparticles possess significant antibiofilm properties [110]. In addition, modifying the surface of nanoparticles with appropriate capping agents can enhance their interaction with biofilms [86]. When nanoparticles (NPs) are introduced into physiological fluids, they become coated by proteins, forming a protein corona (PC) that modifies their physicochemical properties, including their size, charge, and functionality. Recent studies have shown that modifying NPs with an organic corona can improve their interaction with biofilms [111]. Nanotechnology provides significant benefits in treating UTIs by enabling controlled and sustained drug release. When combined with magnetic nanoparticles, macrolides—the primary drugs used to treat biofilm-related infections—exhibit significantly enhanced antimicrobial activity [112]. Table 4 displays the nanoparticles that have demonstrated effectiveness against biofilm-forming bacteria that cause UTIs.

Metal nanoparticles have been shown to inhibit biofilm formation by bacteria that cause UTIs through various mechanisms, such as by damaging bacterial DNA or cell membranes, causing component leakage, and oxidizing constituents by generating reactive oxygen species (ROSs) [128]. In addition to drug delivery, many NPs can be used to impregnate medical devices due to their biocompatibility and innate antimicrobial activity that prevents the formation of biofilms [132].

### 6.4. Bacteriophage Therapy for Treating UTIs

In recent years, there has been renewed interest in bacteriophages as potential agents for combatting antibiotic-resistant and chronic UTIs [133]. Bacteriophages are highly specific in targeting bacteria, safe (as they do not infect eukaryotic cells), lack bacterial resistance mechanisms, and have the potential for easy incorporation into hydrogel-coated catheters [134]. The use of bacteriophages as agents to destroy bacterial biofilm is, therefore, a promising resource. However, the broader use of phage therapy in humans has been limited by the requirement for specific authorizations [135].

### 6.5. Biofilm-Dispersing Enzymes

One of the most promising strategies for biofilm eradication is the search for the enzymatic degradation of EPSs, which account for up to 90% of the total biofilm. Microbial-biofilm-disrupting enzymes belong to three classes: glycosidic hydrolases, deoxyribonucleases, and proteases. They cause the detachment of sessile bacterial cells, making the biofilm more susceptible to antibiotics. Enzymatic biofilm disruption may improve drug access by disrupting biofilm maturation, but issues of biofilm-degrading enzyme toxicity, tolerability, and stability are not fully resolved. In addition, although preclinical studies on enzymatic biofilm dispersion are promising, only limited demonstrations of the safety and efficacy of enzymes in in vivo models are currently available.

## 7. Discussion and Conclusions

Among the infectious diseases associated with the presence of biofilm, those of the urinary tract are especially important. Although the urinary tract has natural antimicrobial host defences, such as urine flow, urinary pH, polymorphonucleate-mediated inflammatory response, and the presence of bacterial adhesion inhibitors, most uropathogens are able to attach to, colonize, and form biofilms in the urinary tract due to the presence of several virulence factors, including adhesins. Over the last two decades, the frequent use of antibiotics for the treatment of infections associated with biofilms has led to a steady and progressive increase in virulent and antibiotic-resistant bacteria that cannot be eradicated with traditional antibiotic treatment [6]. In biofilms, resistance to conventional antimicrobials is mainly due to the inability of these molecules to reach the persister cells within the biofilm. These cells are also resistant to antimicrobials due to their low metabolic activity [16]. They are not mutants, but rather phenotypic variants of the wild type that become active and rebuild the biofilm when antibiotic treatment is stopped. Previous research has demonstrated that biofilm can impede the effectiveness of antimicrobial agents by either obstructing or delaying their spread or chemically interacting with the antibiotics [46]. The high levels of antibiotic resistance that characterise biofilms are largely due to the exopolysaccharide matrix, which represents one of the key elements in the establishment and maintenance of a biofilm’s integrity. For example, lysing alginate, the main component of the extracellular matrix of the biofilm formed by *P. aeruginosa*, hinders the diffusion of antimicrobial substances, suggesting that the matrix polysaccharide of the biofilm is responsible for the resistance of microorganisms to antimicrobial agents [136]. In addition, the level of resistance is also dependent on the stage of development of the biofilm. Studies on *P. aeruginosa* biofilms at different stages have shown that older, slow-growing biofilms are considerably more resistant to antimicrobial agents than younger, fast-growing ones [137]. Due to the resistance of the biofilm to destruction by a single agent, combination therapy has been proposed in several studies to improve the penetration of existing antimicrobial agents into the rigid structure of the biofilm. For example, the combination of clarithromycin, a macrolide antibiotic, and vancomycin was found to completely eradicate a biofilm formed by *P. aeruginosa* and *Staphylococcus* spp. and, thereby, resolve the infection [46]. Bacterial biofilms are the leading cause of healthcare-associated infections in humans, particularly catheter-associated urinary tractitis. While many antibacterial coatings have been developed to prevent biofilm formation on catheter surfaces, including metal ions, nanoparticles, bacteriophages, quorum sensing inhibitors, and bioactive molecules, only a few antifouling coatings have been approved for marketing. Therefore, there is an urgent need for new approaches and strategies for inhibiting biofilm formation.

## 8. Future Directions

Biofilms pose a significant concern in medical-device-associated infections. Currently, many efforts are underway to discover new potential candidates to treat or prevent bacteria that form biofilms and cause UTIs. Although natural and synthetic agents, as well as nanotechnology-based approaches, have shown encouraging antibiofilm activity, their effectiveness is limited. Difficulties in understanding the mechanisms by which these compounds exert their effects on biofilm may explain these limitations. Little is known about the molecular mechanisms that are activated following the interaction of these agents with antibiofilm activity, including signalling cascades, pili gene regulation, efflux pump activity, and altering the EPS structure. In this context, it is important to underline that the gap between in vitro and in vivo testing has not yet been bridged. Despite considerable interest in various agents that inhibit biofilms in vitro, the current models used to test these agents are unable to recapitulate the conditions of the human bladder, where bacterial biofilm formation occurs. This is because in vitro studies cannot perfectly replicate in vivo conditions. As we gain a better understanding of the antibiotic tolerance mechanisms of biofilms, our ability to develop drugs that can overcome bacterial biofilms will improve. It is important to study CAUTIs and their different treatment modalities, bearing in mind that there are differences that cannot be ignored. One possible approach to enhancing the effectiveness of antibiotics against biofilms is to use various combinations of natural and synthetic agents with antibiotics.

## Figures and Tables

**Table 1:** Signs and symptoms of UTIs in different parts of the urinary system.

Organs of Urinary Tract	Signs and Symptoms
Bladder	Dysuria *, blood in urine, frequency *, suprapubic pain
Urethra	Burning with urination, discharge
Kidneys	Nausea, vomiting, high fever, back or side pain
Urethritis	Dysuria *, itching, frequency *

* These symptoms may overlap with the clinical presentation of STIs.

**Table 2:** Antibiofilm activity of antimicrobial peptides (AMPs) against bacteria responsible for UTIs.

AMPs	Antibiofilm Activity	Mechanism of Action	Reference
Nisin A, Mastoparan	*S. aureus*	Membrane depolarisation	[89]
A3	*E. faecalis*, *S. aureus*	Membrane disruption	[90]
Coprisin	*E. coli*, *S. aureus*	Membrane disruption	[91]
GHaK	*S. aureus*	Membrane permeabilisation	[92]
PS1	*P. aeruginossa*, *S. aureus*	EPS production inhibition	[93]
DJK 5/6	*E. coli*, *P. aeruginosa*, *K. pneumoniae*	Cell signal interruption for biofilm formation	[94]
Melittin	*E. coli*, *P. aeruginosa*, *K. pneumoniae*	Membrane permeabilisation	[95]
LL-37	*P. aeruginosa*, *S. epidermidis*	Preventing the transcription of specific genes necessary for quorum sensing	[96]
Hepcidin	*S. epidermidis*	Inhibition of EPS production	[97]

A3: antimicrobial peptide-A3; LL-37: human cathelicidin antimicrobial peptide; temporin-GHa (cloned from Hylarana guentheri); PS1: synthetic peptides.

**Table 3:** Quorum-sensing inhibitors.

QS Inhibitors	Antibiofilm Activity	Mechanism of Action	Reference
Allicin	*P. aeruginosa*, *P. mirabilis*	QS inhibition	[103,104]
5-Hydroxymethylfurfural	*P. aeruginosa*	Downregulation of the expression of quorum-sensing genes	[105]
Glycyrin and glyzarin	*Acinetobacter baumannii*	Inhibition of microbial-quorum-sensing-mediated virulence factors	[106]
Tannic acid	*P. mirabilis* *S. typhi* and *S. paratyphi A*	Restriction of QS-regulated virulence factors	[107,108]

**Table 4:** Antimicrobial action of different NPs against biofilm-forming bacteria that cause UTIs.

NPs	Anti-Biofilm Activity	MIC	Mechanism of Action	Reference
Silver nanoparticles (AgNPs)	*S. aureus*, *E. coli*, *P. aeruginosa*, *P. vulgaris*	0.625 mg/mL (*S. aureus*)	Nano-based drug delivery	[113,114,115]
Fluoride-based nanoparticles	*E. faecalis*, *S. aureus*	0.1 mg/mL (*S. aureus*)	Inhibition of bacterial metabolism	[116,117,118,119]
Polymeric nanoparticles (PNs)	*Gram-positive* and *Gram-negative bacteria*	0.340 mg/mL (*S. aureus*)	Controlled drug delivery	[120,121,122]
Zinc-based nanoparticles	*E. coli*, *S. aureus*	0.05 mg/mL (*S. aureus*)	Disruption of membrane integrity	[116,123]
Gold nanoparticles (AuNPs)	*P. aeruginossa*, *E. coli*, *S. aureus*	7.56 μg/mL (*S. aureus*)	Targeted drug delivery	[124,125,126]
Iron, aluminium oxide, copper oxide, gallium-based NPs	Gram-positive and Gram-negative bacteria	100 μM (*S. aureus*)	ROS generation	[127,128,129,130,131]

## Data Availability

Not applicable.

## Abbreviations

ABC	ATP-binding cassette
AmpC	Cephalosporinase
AMPs	antimicrobial peptides
A3	antimicrobial peptide-A3
CAUTI	catheter-associated urinary tract infections
CDC	Centers for Disease Control and Prevention
CTX-M	Cefotaximase
EPS	Exopolysaccharides
GAG layer	Glycosaminoglycan
HGT	horizontal gene transfer
KPC	carbapenemase-producing *Klebsiella pneumoniae*
LL-37	human cathelicidin antimicrobial peptide
MRSA	methicillin-resistant *Staphylococcus aureus*
NDM	New Delhi Metallo β-lattamasi
PS1	synthetic peptides
OXA	oxacillinase
SHV	sulfhydryl variant of TEM
TEM	β-lactamase isolated from a patient named Temoniera in Greece
temporinGHa	cloned from Hylarana guentheri
UPEC	uropathogenic *Escherichia coli*
UTIs	urinary tract infections
VRE	vancomycin-resistant *Enterococcus*

## References

[1] Maddali N., Cantin A., Koshy S., Eiting E., Fedorenko M. (2021). Antibiotic prescribing patterns for adult urinary tract infections within emergency department and urgent care settings. Am. J. Emerg. Med..

[2] Brodie A., El-Taji O., Jour I., Foley C., Hanbury D. (2020). A Retrospective Study of Immunotherapy Treatment with Uro-Vaxom (OM-89(R)) for Prophylaxis of Recurrent Urinary Tract Infections. Curr. Urol..

[3] Medina M., Castillo-Pino E. (2019). An introduction to the epidemiology and burden of urinary tract infections. Ther. Adv. Urol..

[4] Flores-Mireles A.L., Walker J.N., Caparon M., Hultgren S.J. (2015). Urinary tract infections: Epidemiology, mechanisms of infection and treatment options. Nat. Rev. Microbiol..

[5] Biondo C. (2023). New Insights into the Pathogenesis and Treatment of Urinary Tract Infections. Pathogens.

[6] Mancuso G., Midiri A., Gerace E., Marra M., Zummo S., Biondo C. (2023). Urinary Tract Infections: The Current Scenario and Future Prospects. Pathogens.

[7] Goebel M.C., Trautner B.W., Grigoryan L. (2021). The Five Ds of Outpatient Antibiotic Stewardship for Urinary Tract Infections. Clin. Microbiol. Rev..

[8] Dadgostar P. (2019). Antimicrobial Resistance: Implications and Costs. Infect. Drug Resist..

[9] Paul R. (2018). State of the Globe: Rising Antimicrobial Resistance of Pathogens in Urinary Tract Infection. J. Glob. Infect. Dis..

[10] Walsh T.R., Gales A.C., Laxminarayan R., Dodd P.C. (2023). Antimicrobial Resistance: Addressing a Global Threat to Humanity. PLoS Med..

[11] Mancuso G., Midiri A., Zummo S., Gerace E., Scappatura G., Biondo C. (2021). Extended-spectrum beta-lactamase & carbapenemase-producing fermentative Gram-negative bacilli in clinical isolates from a University Hospital in Southern Italy. New Microbiol..

[12] Li X., Fan H., Zi H., Hu H., Li B., Huang J., Luo P., Zeng X. (2022). Global and Regional Burden of Bacterial Antimicrobial Resistance in Urinary Tract Infections in 2019. J. Clin. Med..

[13] Antimicrobial Resistance C. (2022). Global burden of bacterial antimicrobial resistance in 2019: A systematic analysis. Lancet.

[14] Mancuso G., Midiri A., Gerace E., Biondo C. (2021). Bacterial Antibiotic Resistance: The Most Critical Pathogens. Pathogens.

[15] Mlugu E.M., Mohamedi J.A., Sangeda R.Z., Mwambete K.D. (2023). Prevalence of urinary tract infection and antimicrobial resistance patterns of uropathogens with biofilm forming capacity among outpatients in morogoro, Tanzania: A cross-sectional study. BMC Infect. Dis..

[16] Uruen C., Chopo-Escuin G., Tommassen J., Mainar-Jaime R.C., Arenas J. (2020). Biofilms as Promoters of Bacterial Antibiotic Resistance and Tolerance. Antibiotics.

[17] Vestby L.K., Gronseth T., Simm R., Nesse L.L. (2020). Bacterial Biofilm and its Role in the Pathogenesis of Disease. Antibiotics.

[18] Lila A.S.A., Rajab A.A.H., Abdallah M.H., Rizvi S.M.D., Moin A., Khafagy E.S., Tabrez S., Hegazy W.A.H. (2023). Biofilm Lifestyle in Recurrent Urinary Tract Infections. Life.

[19] Hola V., Opazo-Capurro A., Scavone P. (2021). Editorial: The Biofilm Lifestyle of Uropathogens. Front. Cell. Infect. Microbiol..

[20] Tan C.W., Chlebicki M.P. (2016). Urinary tract infections in adults. Singap. Med. J..

[21] Sihra N., Goodman A., Zakri R., Sahai A., Malde S. (2018). Nonantibiotic prevention and management of recurrent urinary tract infection. Nat. Rev. Urol..

[22] Li J., Yu Y.F., Qi X.W., Du Y., Li C.Q. (2022). Immune-related ureteritis and cystitis induced by immune checkpoint inhibitors: Case report and literature review. Front. Immunol..

[23] Mahyar A., Ayazi P., Farzadmanesh S., Sahmani M., Oveisi S., Chegini V., Esmaeily S. (2015). The role of zinc in acute pyelonephritis. Infez. Med..

[24] Van Eyssen S.R., Samarkina A., Isbilen O., Zeden M.S., Volkan E. (2023). FimH and Type 1 Pili Mediated Tumor Cell Cytotoxicity by Uropathogenic *Escherichia coli* In Vitro. Pathogens.

[25] Alelign T., Petros B. (2018). Kidney Stone Disease: An Update on Current Concepts. Adv. Urol..

[26] Werneburg G.T. (2022). Catheter-Associated Urinary Tract Infections: Current Challenges and Future Prospects. Res. Rep. Urol..

[27] Ingram A., Posid T., Pandit A., Rose J., Amin S., Bellows F. (2021). Risk factors, demographic profiles, and management of uncomplicated recurrent urinary tract infections: A single institution study. Menopause.

[28] Zare M., Vehreschild M., Wagenlehner F. (2022). Management of uncomplicated recurrent urinary tract infections. BJU Int..

[29] Mekonnen S.A., El Husseini N., Turdiev A., Carter J.A., Belew A.T., El-Sayed N.M., Lee V.T. (2022). Catheter-associated urinary tract infection by *Pseudomonas aeruginosa* progresses through acute and chronic phases of infection. Proc. Natl. Acad. Sci. USA.

[30] Guliciuc M., Porav-Hodade D., Mihailov R., Rebegea L.F., Voidazan S.T., Ghirca V.M., Maier A.C., Marinescu M., Firescu D. (2023). Exploring the Dynamic Role of Bacterial Etiology in Complicated Urinary Tract Infections. Medicina.

[31] Woodford H.J., George J. (2011). Diagnosis and management of urinary infections in older people. Clin. Med..

[32] Geerlings S.E. (2016). Clinical Presentations and Epidemiology of Urinary Tract Infections. Microbiol. Spectr..

[33] Ebell M.H., Gagyor I. (2022). Diagnosis of Urinary Tract Infection in Women. Am. Fam. Physician.

[34] Kwok M., McGeorge S., Mayer-Coverdale J., Graves B., Paterson D.L., Harris P.N.A., Esler R., Dowling C., Britton S., Roberts M.J. (2022). Guideline of guidelines: Management of recurrent urinary tract infections in women. BJU Int..

[35] Caretto M., Giannini A., Russo E., Simoncini T. (2017). Preventing urinary tract infections after menopause without antibiotics. Maturitas.

[36] Alperin M., Burnett L., Lukacz E., Brubaker L. (2019). The mysteries of menopause and urogynecologic health: Clinical and scientific gaps. Menopause.

[37] Lipsky B.A., Byren I., Hoey C.T. (2010). Treatment of bacterial prostatitis. Clin. Infect. Dis..

[38] Le B., Schaeffer A.J. (2011). Chronic prostatitis. BMJ Clin. Evid..

[39] Sell J., Nasir M., Courchesne C. (2021). Urethritis: Rapid Evidence Review. Am. Fam. Physician.

[40] Sadoghi B., Kranke B., Komericki P., Hutterer G. (2022). Sexually transmitted pathogens causing urethritis: A mini-review and proposal of a clinically based diagnostic and therapeutic algorithm. Front. Med..

[41] Herness J., Buttolph A., Hammer N.C. (2020). Acute Pyelonephritis in Adults: Rapid Evidence Review. Am. Fam. Physician.

[42] Kline K.A., Lewis A.L. (2016). Gram-Positive Uropathogens, Polymicrobial Urinary Tract Infection, and the Emerging Microbiota of the Urinary Tract. Microbiol. Spectr..

[43] Roth R.S., Liden M., Huttner A. (2023). The urobiome in men and women: A clinical review. Clin. Microbiol. Infect..

[44] Colella M., Topi S., Palmirotta R., D’Agostino D., Charitos I.A., Lovero R., Santacroce L. (2023). An Overview of the Microbiota of the Human Urinary Tract in Health and Disease: Current Issues and Perspectives. Life.

[45] Govindarajan D.K., Kandaswamy K. (2022). Virulence factors of uropathogens and their role in host pathogen interactions. Cell Surf..

[46] Sharma S., Mohler J., Mahajan S.D., Schwartz S.A., Bruggemann L., Aalinkeel R. (2023). Microbial Biofilm: A Review on Formation, Infection, Antibiotic Resistance, Control Measures, and Innovative Treatment. Microorganisms.

[47] Qin S., Xiao W., Zhou C., Pu Q., Deng X., Lan L., Liang H., Song X., Wu M. (2022). *Pseudomonas aeruginosa*: Pathogenesis, virulence factors, antibiotic resistance, interaction with host, technology advances and emerging therapeutics. Signal Transduct. Target. Ther..

[48] Flores-Diaz M., Monturiol-Gross L., Naylor C., Alape-Giron A., Flieger A. (2016). Bacterial Sphingomyelinases and Phospholipases as Virulence Factors. Microbiol. Mol. Biol. Rev..

[49] Singh V., Phukan U.J. (2019). Interaction of host and *Staphylococcus aureus* protease-system regulates virulence and pathogenicity. Med. Microbiol. Immunol..

[50] Ramirez-Larrota J.S., Eckhard U. (2022). An Introduction to Bacterial Biofilms and Their Proteases, and Their Roles in Host Infection and Immune Evasion. Biomolecules.

[51] Whelan S., Lucey B., Finn K. (2023). Uropathogenic *Escherichia coli* (UPEC)-Associated Urinary Tract Infections: The Molecular Basis for Challenges to Effective Treatment. Microorganisms.

[52] Sarshar M., Behzadi P., Ambrosi C., Zagaglia C., Palamara A.T., Scribano D. (2020). FimH and Anti-Adhesive Therapeutics: A Disarming Strategy against Uropathogens. Antibiotics.

[53] Assefa M., Amare A. (2022). Biofilm-Associated Multi-Drug Resistance in Hospital-Acquired Infections: A Review. Infect. Drug Resist..

[54] Soto S.M. (2014). Importance of Biofilms in Urinary Tract Infections: New Therapeutic Approaches. Adv. Biol..

[55] Zhang K., Li X., Yu C., Wang Y. (2020). Promising Therapeutic Strategies against Microbial Biofilm Challenges. Front. Cell. Infect. Microbiol..

[56] Di Martino P. (2018). Extracellular polymeric substances, a key element in understanding biofilm phenotype. AIMS Microbiol..

[57] Guzman-Soto I., McTiernan C., Gonzalez-Gomez M., Ross A., Gupta K., Suuronen E.J., Mah T.F., Griffith M., Alarcon E.I. (2021). Mimicking biofilm formation and development: Recent progress in in vitro and in vivo biofilm models. iScience.

[58] Singh S., Datta S., Narayanan K.B., Rajnish K.N. (2021). Bacterial exo-polysaccharides in biofilms: Role in antimicrobial resistance and treatments. J. Genet. Eng. Biotechnol..

[59] Abdalla A.K., Ayyash M.M., Olaimat A.N., Osaili T.M., Al-Nabulsi A.A., Shah N.P., Holley R. (2021). Exopolysaccharides as Antimicrobial Agents: Mechanism and Spectrum of Activity. Front. Microbiol..

[60] Rather M.A., Gupta K., Mandal M. (2021). Microbial biofilm: Formation, architecture, antibiotic resistance, and control strategies. Braz. J. Microbiol..

[61] Li Y., Li X., Hao Y., Liu Y., Dong Z., Li K. (2021). Biological and Physiochemical Methods of Biofilm Adhesion Resistance Control of Medical-Context Surface. Int. J. Biol. Sci..

[62] Oluwole O.M. (2022). Biofilm: Formation and Natural Products’ Approach to Control—A Review. Afr. J. Infect. Dis..

[63] Costa O.Y.A., Raaijmakers J.M., Kuramae E.E. (2018). Microbial Extracellular Polymeric Substances: Ecological Function and Impact on Soil Aggregation. Front. Microbiol..

[64] Balducci E., Papi F., Capialbi D.E., Del Bino L. (2023). Polysaccharides’ Structures and Functions in Biofilm Architecture of Antimicrobial-Resistant (AMR) Pathogens. Int. J. Mol. Sci..

[65] Preda V.G., Sandulescu O. (2019). Communication is the key: Biofilms, quorum sensing, formation and prevention. Discoveries.

[66] Fu J., Zhang Y., Lin S., Zhang W., Shu G., Lin J., Li H., Xu F., Tang H., Peng G. (2021). Strategies for Interfering with Bacterial Early Stage Biofilms. Front. Microbiol..

[67] Sharma K., Dhar N., Thacker V.V., Simonet T.M., Signorino-Gelo F., Knott G.W., McKinney J.D. (2021). Dynamic persistence of UPEC intracellular bacterial communities in a human bladder-chip model of urinary tract infection. eLife.

[68] Szabo S., Feier B., Capatina D., Tertis M., Cristea C., Popa A. (2022). An Overview of Healthcare Associated Infections and Their Detection Methods Caused by Pathogen Bacteria in Romania and Europe. J. Clin. Med..

[69] Rubi H., Mudey G., Kunjalwar R. (2022). Catheter-Associated Urinary Tract Infection (CAUTI). Cureus.

[70] Yuan F., Huang Z., Yang T., Wang G., Li P., Yang B., Li J. (2021). Pathogenesis of Proteus mirabilis in Catheter-Associated Urinary Tract Infections. Urol. Int..

[71] Murray B.O., Flores C., Williams C., Flusberg D.A., Marr E.E., Kwiatkowska K.M., Charest J.L., Isenberg B.C., Rohn J.L. (2021). Recurrent Urinary Tract Infection: A Mystery in Search of Better Model Systems. Front. Cell. Infect. Microbiol..

[72] Terlizzi M.E., Gribaudo G., Maffei M.E. (2017). UroPathogenic *Escherichia coli* (UPEC) Infections: Virulence Factors, Bladder Responses, Antibiotic, and Non-antibiotic Antimicrobial Strategies. Front. Microbiol..

[73] Khan J., Tarar S.M., Gul I., Nawaz U., Arshad M. (2021). Challenges of antibiotic resistance biofilms and potential combating strategies: A review. 3 Biotech.

[74] Abdelhamid A.G., Yousef A.E. (2023). Combating Bacterial Biofilms: Current and Emerging Antibiofilm Strategies for Treating Persistent Infections. Antibiotics.

[75] Michaelis C., Grohmann E. (2023). Horizontal Gene Transfer of Antibiotic Resistance Genes in Biofilms. Antibiotics.

[76] Tao S., Chen H., Li N., Wang T., Liang W. (2022). The Spread of Antibiotic Resistance Genes In Vivo Model. Can. J. Infect. Dis. Med. Microbiol..

[77] Palusiak A. (2022). Proteus mirabilis and *Klebsiella pneumoniae* as pathogens capable of causing co-infections and exhibiting similarities in their virulence factors. Front. Cell. Infect. Microbiol..

[78] Cortese Y.J., Wagner V.E., Tierney M., Devine D., Fogarty A. (2018). Review of Catheter-Associated Urinary Tract Infections and In Vitro Urinary Tract Models. J. Healthc. Eng..

[79] Kanti S.P.Y., Csoka I., Jojart-Laczkovich O., Adalbert L. (2022). Recent Advances in Antimicrobial Coatings and Material Modification Strategies for Preventing Urinary Catheter-Associated Complications. Biomedicines.

[80] Stahlhut S.G., Struve C., Krogfelt K.A., Reisner A. (2012). Biofilm formation of *Klebsiella pneumoniae* on urethral catheters requires either type 1 or type 3 fimbriae. FEMS Immunol. Med. Microbiol..

[81] Taha H., Raji S.J., Khallaf A., Abu Hija S., Mathew R., Rashed H., Du Plessis C., Allie Z., Ellahham S. (2017). Improving Catheter Associated Urinary Tract Infection Rates in the Medical Units. BMJ Qual. Improv. Rep..

[82] Van Decker S.G., Bosch N., Murphy J. (2021). Catheter-associated urinary tract infection reduction in critical care units: A bundled care model. BMJ Open Qual..

[83] Shrestha L.B., Baral R., Khanal B. (2019). Comparative study of antimicrobial resistance and biofilm formation among Gram-positive uropathogens isolated from community-acquired urinary tract infections and catheter-associated urinary tract infections. Infect. Drug Resist..

[84] Verderosa A.D., Totsika M., Fairfull-Smith K.E. (2019). Bacterial Biofilm Eradication Agents: A Current Review. Front. Chem..

[85] Mirghani R., Saba T., Khaliq H., Mitchell J., Do L., Chambi L., Diaz K., Kennedy T., Alkassab K., Huynh T. (2022). Biofilms: Formation, drug resistance and alternatives to conventional approaches. AIMS Microbiol..

[86] Sahli C., Moya S.E., Lomas J.S., Gravier-Pelletier C., Briandet R., Hemadi M. (2022). Recent advances in nanotechnology for eradicating bacterial biofilm. Theranostics.

[87] Lopes B.S., Hanafiah A., Nachimuthu R., Muthupandian S., Md Nesran Z.N., Patil S. (2022). The Role of Antimicrobial Peptides as Antimicrobial and Antibiofilm Agents in Tackling the Silent Pandemic of Antimicrobial Resistance. Molecules.

[88] Pontes J.T.C., Toledo Borges A.B., Roque-Borda C.A., Pavan F.R. (2022). Antimicrobial Peptides as an Alternative for the Eradication of Bacterial Biofilms of Multi-Drug Resistant Bacteria. Pharmaceutics.

[89] Okuda K., Zendo T., Sugimoto S., Iwase T., Tajima A., Yamada S., Sonomoto K., Mizunoe Y. (2013). Effects of bacteriocins on methicillin-resistant *Staphylococcus aureus* biofilm. Antimicrob. Agents Chemother..

[90] Almaaytah A., Farajallah A., Abualhaijaa A., Al-Balas Q. (2018). A3, a Scorpion Venom Derived Peptide Analogue with Potent Antimicrobial and Potential Antibiofilm Activity against Clinical Isolates of Multi-Drug Resistant Gram Positive Bacteria. Molecules.

[91] Hwang I.S., Hwang J.S., Hwang J.H., Choi H., Lee E., Kim Y., Lee D.G. (2013). Synergistic effect and antibiofilm activity between the antimicrobial peptide coprisin and conventional antibiotics against opportunistic bacteria. Curr. Microbiol..

[92] Xie Z., Wei H., Meng J., Cheng T., Song Y., Wang M., Zhang Y. (2019). The Analogs of Temporin-GHa Exhibit a Broader Spectrum of Antimicrobial Activity and a Stronger Antibiofilm Potential against *Staphylococcus aureus*. Molecules.

[93] Park S.C., Lee M.Y., Kim J.Y., Kim H., Jung M., Shin M.K., Lee W.K., Cheong G.W., Lee J.R., Jang M.K. (2019). Anti-Biofilm Effects of Synthetic Antimicrobial Peptides Against Drug-Resistant *Pseudomonas aeruginosa* and *Staphylococcus aureus* Planktonic Cells and Biofilm. Molecules.

[94] de la Fuente-Nunez C., Cardoso M.H., de Souza Candido E., Franco O.L., Hancock R.E. (2016). Synthetic antibiofilm peptides. Biochim. Biophys. Acta.

[95] Dosler S., Karaaslan E., Alev Gerceker A. (2016). Antibacterial and anti-biofilm activities of melittin and colistin, alone and in combination with antibiotics against Gram-negative bacteria. J. Chemother..

[96] de la Fuente-Nunez C., Korolik V., Bains M., Nguyen U., Breidenstein E.B., Horsman S., Lewenza S., Burrows L., Hancock R.E. (2012). Inhibition of bacterial biofilm formation and swarming motility by a small synthetic cationic peptide. Antimicrob. Agents Chemother..

[97] Batoni G., Maisetta G., Esin S. (2016). Antimicrobial peptides and their interaction with biofilms of medically relevant bacteria. Biochim. Biophys. Acta.

[98] Moreno-Gamez S., Hochberg M.E., van Doorn G.S. (2023). Quorum sensing as a mechanism to harness the wisdom of the crowds. Nat. Commun..

[99] Singh S., Bhatia S. (2021). Quorum Sensing Inhibitors: Curbing Pathogenic Infections through Inhibition of Bacterial Communication. Iran. J. Pharm. Res..

[100] Duplantier M., Lohou E., Sonnet P. (2021). Quorum Sensing Inhibitors to Quench *P. aeruginosa* Pathogenicity. Pharmaceuticals.

[101] Mishra R., Panda A.K., De Mandal S., Shakeel M., Bisht S.S., Khan J. (2020). Natural Anti-biofilm Agents: Strategies to Control Biofilm-Forming Pathogens. Front. Microbiol..

[102] Khameneh B., Eskin N.A.M., Iranshahy M., Fazly Bazzaz B.S. (2021). Phytochemicals: A Promising Weapon in the Arsenal against Antibiotic-Resistant Bacteria. Antibiotics.

[103] Imani Rad H., Peeri H., Amani M., Mohammadnia A., Ogunniyi A.D., Khazandi M., Venter H., Arzanlou M. (2019). Allicin prevents the formation of Proteus-induced urinary crystals and the blockage of catheter in a bladder model in vitro. Microb. Pathog..

[104] Jakobsen T.H., Warming A.N., Vejborg R.M., Moscoso J.A., Stegger M., Lorenzen F., Rybtke M., Andersen J.B., Petersen R., Andersen P.S. (2017). A broad range quorum sensing inhibitor working through sRNA inhibition. Sci. Rep..

[105] Rajkumari J., Borkotoky S., Reddy D., Mohanty S.K., Kumavath R., Murali A., Suchiang K., Busi S. (2019). Anti-quorum sensing and anti-biofilm activity of 5-hydroxymethylfurfural against *Pseudomonas aeruginosa* PAO1: Insights from in vitro, in vivo and in silico studies. Microbiol. Res..

[106] Bhargava N., Singh S.P., Sharma A., Sharma P., Capalash N. (2015). Attenuation of quorum sensing-mediated virulence of Acinetobacter baumannii by Glycyrrhiza glabra flavonoids. Future Microbiol..

[107] Sivasankar C., Jha N.K., Ghosh R., Shetty P.H. (2020). Anti quorum sensing and anti virulence activity of tannic acid and it’s potential to breach resistance in Salmonella enterica Typhi/Paratyphi A clinical isolates. Microb. Pathog..

[108] Jones S.M., Dang T.T., Martinuzzi R. (2009). Use of quorum sensing antagonists to deter the formation of crystalline Proteus mirabilis biofilms. Int. J. Antimicrob. Agents.

[109] Crintea A., Carpa R., Mitre A.O., Petho R.I., Chelaru V.F., Nadasan S.M., Neamti L., Dutu A.G. (2023). Nanotechnology Involved in Treating Urinary Tract Infections: An Overview. Nanomaterials.

[110] Moradi F., Ghaedi A., Fooladfar Z., Bazrgar A. (2023). Recent advance on nanoparticles or nanomaterials with anti-multidrug resistant bacteria and anti-bacterial biofilm properties: A systematic review. Heliyon.

[111] Garcia Vence M., Chantada-Vazquez M.D.P., Vazquez-Estevez S., Manuel Cameselle-Teijeiro J., Bravo S.B., Nunez C. (2020). Potential clinical applications of the personalized, disease-specific protein corona on nanoparticles. Clin. Chim. Acta Int. J. Clin. Chem..

[112] Siraj E.A., Yayehrad A.T., Belete A. (2023). How Combined Macrolide Nanomaterials are Effective against Resistant Pathogens? A Comprehensive Review of the Literature. Int. J. Nanomed..

[113] Bharadwaj K.K., Rabha B., Pati S., Choudhury B.K., Sarkar T., Gogoi S.K., Kakati N., Baishya D., Kari Z.A., Edinur H.A. (2021). Green Synthesis of Silver Nanoparticles Using Diospyros malabarica Fruit Extract and Assessments of Their Antimicrobial, Anticancer and Catalytic Reduction of 4-Nitrophenol (4-NP). Nanomaterials.

[114] Palanisamy N.K., Ferina N., Amirulhusni A.N., Mohd-Zain Z., Hussaini J., Ping L.J., Durairaj R. (2014). Antibiofilm properties of chemically synthesized silver nanoparticles found against *Pseudomonas aeruginosa*. J. Nanobiotechnol..

[115] Ravindran D., Ramanathan S., Arunachalam K., Jeyaraj G.P., Shunmugiah K.P., Arumugam V.R. (2018). Phytosynthesized silver nanoparticles as antiquorum sensing and antibiofilm agent against the nosocomial pathogen Serratia marcescens: An in vitro study. J. Appl. Microbiol..

[116] Shunmugaperumal T., Ramamurthy S. (2012). Assessment of antibiofilm activity of magnesium fluoride nanoparticles-stabilized oil-in-water nanosized emulsion. Drug Dev. Ind. Pharm..

[117] Lellouche J., Kahana E., Elias S., Gedanken A., Banin E. (2009). Antibiofilm activity of nanosized magnesium fluoride. Biomaterials.

[118] Guha-Chowdhury N., Clark A.G., Sissons C.H. (1997). Inhibition of purified enolases from oral bacteria by fluoride. Oral Microbiol. Immunol..

[119] Lellouche J., Friedman A., Gedanken A., Banin E. (2012). Antibacterial and antibiofilm properties of yttrium fluoride nanoparticles. Int. J. Nanomed..

[120] Dos Santos Ramos M.A., Da Silva P.B., Sposito L., De Toledo L.G., Bonifacio B.V., Rodero C.F., Dos Santos K.C., Chorilli M., Bauab T.M. (2018). Nanotechnology-based drug delivery systems for control of microbial biofilms: A review. Int. J. Nanomed..

[121] Rabha B., Bharadwaj K.K., Baishya D., Sarkar T., Edinur H.A., Pati S. (2021). Synthesis and Characterization of Diosgenin Encapsulated Poly-epsilon-Caprolactone-Pluronic Nanoparticles and Its Effect on Brain Cancer Cells. Polymers.

[122] Liu S., Qiao S., Li L., Qi G., Lin Y., Qiao Z., Wang H., Shao C. (2015). Surface charge-conversion polymeric nanoparticles for photodynamic treatment of urinary tract bacterial infections. Nanotechnology.

[123] Abd Elkodous M., El-Sayyad G.S., Abdel Maksoud M.I.A., Abdelrahman I.Y., Mosallam F.M., Gobara M., El-Batal A.I. (2020). Fabrication of Ultra-Pure Anisotropic Zinc Oxide Nanoparticles via Simple and Cost-Effective Route: Implications for UTI and EAC Medications. Biol. Trace Elem. Res..

[124] Singh P., Pandit S., Beshay M., Mokkapati V., Garnaes J., Olsson M.E., Sultan A., Mackevica A., Mateiu R.V., Lutken H. (2018). Anti-biofilm effects of gold and silver nanoparticles synthesized by the Rhodiola rosea rhizome extracts. Artif. Cells Nanomed. Biotechnol..

[125] Yu K., Lo J.C., Yan M., Yang X., Brooks D.E., Hancock R.E., Lange D., Kizhakkedathu J.N. (2017). Anti-adhesive antimicrobial peptide coating prevents catheter associated infection in a mouse urinary infection model. Biomaterials.

[126] Shamaila S., Zafar N., Riaz S., Sharif R., Nazir J., Naseem S. (2016). Gold Nanoparticles: An Efficient Antimicrobial Agent against Enteric Bacterial Human Pathogen. Nanomaterials.

[127] Alomary M.N., Ansari M.A. (2021). Proanthocyanin-Capped Biogenic TiO(2) Nanoparticles with Enhanced Penetration, Antibacterial and ROS Mediated Inhibition of Bacteria Proliferation and Biofilm Formation: A Comparative Approach. Chemistry.

[128] Qindeel M., Barani M., Rahdar A., Arshad R., Cucchiarini M. (2021). Nanomaterials for the Diagnosis and Treatment of Urinary Tract Infections. Nanomaterials.

[129] Aderibigbe B.A. (2017). Metal-Based Nanoparticles for the Treatment of Infectious Diseases. Molecules.

[130] Agarwala M., Choudhury B., Yadav R.N. (2014). Comparative study of antibiofilm activity of copper oxide and iron oxide nanoparticles against multidrug resistant biofilm forming uropathogens. Indian J. Microbiol..

[131] Li F., Liu F., Huang K., Yang S. (2022). Advancement of Gallium and Gallium-Based Compounds as Antimicrobial Agents. Front. Bioeng. Biotechnol..

[132] Polivkova M., Hubacek T., Staszek M., Svorcik V., Siegel J. (2017). Antimicrobial Treatment of Polymeric Medical Devices by Silver Nanomaterials and Related Technology. Int. J. Mol. Sci..

[133] Zalewska-Piatek B., Piatek R. (2021). Bacteriophages as Potential Tools for Use in Antimicrobial Therapy and Vaccine Development. Pharmaceuticals.

[134] Amankwah S., Abdella K., Kassa T. (2021). Bacterial Biofilm Destruction: A Focused Review on the Recent Use of Phage-Based Strategies with Other Antibiofilm Agents. Nanotechnol. Sci. Appl..

[135] Suh G.A., Lodise T.P., Tamma P.D., Knisely J.M., Alexander J., Aslam S., Barton K.D., Bizzell E., Totten K.M.C., Campbell J.L. (2022). Considerations for the Use of Phage Therapy in Clinical Practice. Antimicrob. Agents Chemother..

[136] Thi M.T.T., Wibowo D., Rehm B.H.A. (2020). *Pseudomonas aeruginosa* Biofilms. Int. J. Mol. Sci..

[137] Ciofu O., Tolker-Nielsen T. (2019). Tolerance and Resistance of *Pseudomonas aeruginosa* Biofilms to Antimicrobial Agents-How *P. aeruginosa* Can Escape Antibiotics. Front. Microbiol..

